# Growth Outcomes and Final Height in Children with Acquired Hypothyroidism: A Systematic Review

**DOI:** 10.3390/children11121510

**Published:** 2024-12-11

**Authors:** Ignazio Cammisa, Donato Rigante, Clelia Cipolla

**Affiliations:** 1 Department of Life Sciences and Public Health, Fondazione Policlinico Universitario A. Gemelli IRCCS, 00168 Rome, Italy; donato.rigante@unicatt.it (D.R.); clelia.cipolla@policlinicogemelli.it (C.C.); 2 Department of Life Sciences and Public Health, Università Cattolica Sacro Cuore, 00168 Rome, Italy

**Keywords:** acquired hypothyroidism, thyroid, final height, pediatric growth, catch-up growth, personalized medicine

## Abstract

Background/Objectives: Hypothyroidism can profoundly affect growth, particularly if it insidiously arises during early childhood. Congenital hypothyroidism is now detected through newborn screening, significantly improving the overall growth outcomes of these children. Conversely, acquired hypothyroidism often results in delayed somatic growth and shorter stature, with many affected children initially remaining non-symptomatic. The main objective of this review is to summarize the current knowledge about the impacts of acquired hypothyroidism on children’s growth outcomes. Methods: We performed a literature review to analyze growth and final height in children with acquired hypothyroidism, matching the following keywords: “hypothyroidism & growth”, “hypothyroidism & height”, “hypothyroidism & stature”, “hypothyroidism & development”, “hypothyroidism & auxological parameters”. We reviewed each article that met the eligibility criteria, and after a thorough selection, we included 16 studies. Results: Growth arrest is frequently noted as a symptom in hypothyroidic children, with substantial portions of affected children presenting below the third percentile for height. The timing of diagnosis significantly influences growth outcomes: those diagnosed during puberty tend to experience less catch-up growth due to accelerated skeletal maturation. Even if thyroxine replacement can induce rapid catch-up growth, it may be incomplete if treatment begins during puberty or if there is a markedly prolonged deficiency of thyroid hormones. While levothyroxine therapy typically results in some degree of catch-up growth, many children do not reach their expected genetic height. Conclusions: This review highlights the necessity of both early diagnosis and treatment of acquired hypothyroidism. Even if many children show improvements in height velocity post-treatment, the complete normalization of growth may remain elusive.

## 1. Introduction

Hypothyroidism is the main prevalent disorder of the thyroid gland in children and adolescents, with significant consequences for growth, development, metabolism, and cognitive functions [1,2]. Primary hypothyroidism is characterized by insufficient thyroid hormone production due to dysfunction of the thyroid gland itself, whereas secondary or central hypothyroidism, which is less common, arises from defects in the pituitary gland or in the hypothalamus [2]. Two main forms of hypothyroidism can be recognized in pediatric patients. The first is congenital hypothyroidism, affecting approximately 1 in 1.500 to 3.000 newborns, typically caused by thyroid dysgenesis, accounting for 80–85% of cases, or by defects in thyroid hormone production and release [2,3]. The second is acquired hypothyroidism, with a prevalence of 1–2%, most often related to autoimmune disorders such as Hashimoto’s thyroiditis, which is its leading cause. However, it can also be related to genetic diseases such as Down syndrome and Turner syndrome, and also to celiac disease or iodine deficiency or radiation exposure [2]. For example, Hashimoto’s thyroiditis has a prevalence of 1.4–52.6% in Down syndrome and 6.3–31% in Turner syndrome [4,5]. Thyroid hormones are essential for growth and development, particularly during childhood, as they have a leading role in skeletal development and the preservation of a normal stature. It is well-established that they act on the primary cells responsible for bone development, promoting both proliferation and differentiation of chondrocytes within the growth plates of long bones, which are crucial for linear growth [6]. Specifically, the active hormone 3,5,3′-l-triiodothyronine (T3) enters target cells via specific transport proteins and binds to thyroid hormone receptor α (TRα), the main receptor in bone and cartilage, mediating T3’s actions [6,7,8,9]. Thyroid hormone deficiency in the acquired form of hypothyroidism, if detected late or left untreated, can result in delayed bone maturation, reduced growth velocity, and short stature [6,7]. Early diagnosis and treatment are crucial to mitigate these effects, as thyroid hormone replacement therapy can promote catch-up growth, ensuring regular growth and harmonious development. The aim of this review is to evaluate growth outcomes and final height in children with acquired hypothyroidism by analyzing the current evidence in the medical literature and assessing the impact of early diagnosis and adequate replacement therapy on catch-up growth.

## 2. Materials and Methods

### 2.1. Data Sources

We carried out a literature review, without date or time limits, to assess growth outcomes and final height in children with acquired hypothyroidism, using five different cues on PubMed with the following keywords: “hypothyroidism & growth”, “hypothyroidism & height”, “hypothyroidism & stature”, “hypothyroidism & development”, “hypothyroidism & auxological parameters”. In order to be deemed eligible for this review, the papers needed to meet the following criteria: (1) children with a diagnosis of acquired hypothyroidism; (2) children who were subjected to auxological evaluation. We excluded the following: (1) non-English language papers; (2) children with a diagnosis of congenital hypothyroidism; (3) studies in which auxological parameters were not assessed according to standardized and validated scales. The abstracts of such papers were assessed by two researchers (IC and CC), who strictly applied the inclusion/exclusion criteria cited above in order to decide whether a paper was eligible for full review. Every paper that satisfied the eligibility criteria was thoroughly analyzed by the authors in its entirety, with any eventual differences of opinion addressed through open discussion.

### 2.2. Study Selection

**Figure 1:**
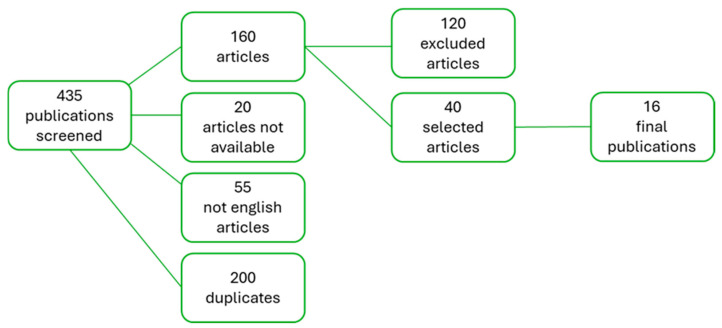
Overview of the process used to select the publications used in this review.

In total, we found 435 records through database searches. The first step involved excluding 55 articles in languages other than English, 20 records with unavailable related articles, and 200 duplicate papers. In the second step, we removed 120 records based on title and abstract evaluation, as they did not meet the inclusion criteria outlined earlier. Of the remaining 40 studies, 24 were excluded after further discussion regarding the reliability of the data. Consequently, 16 articles were selected for inclusion in this review. A detailed overview of the selection process is shown in Figure 1. A wide and extensive summary of all results is shown in Table 1 and Table 2.

### 2.3. Data Extraction

The data extraction template was completed under the close supervision of the principal investigator (CC). The data gathered from each eligible paper included the following: study design, population sample, mean age, sex prevalence, follow-up period, starting time and dose of the replacement therapy, growth and height outcomes. We also examined all the limitations of the studies and any potential conflicts of interest declared by the authors, if mentioned. In this review, we analyzed the ongoing medical literature on the growth outcomes of children with acquired hypothyroidism. Formal ethical approval was not required for our study.

## 3. Results

Among the 16 articles selected in this review [10,11,12,13,14,15,16,17,18,19,20,21,22,23,24,25], 13 primarily aimed to assess growth outcomes in children with acquired hypothyroidism, while 3 articles [13,17,25] were focused on the clinical and laboratory potential effects, including growth measures, of acquired hypothyroidism. The year of publication ranged from 1988 to 2023; most articles were published in the last 15 years (n = 12), emphasizing the significance of our topic in light of recent scientific findings. Out of the 16 selected studies, 4 analyzed growth outcomes in children with subclinical hypothyroidism (Table 2). Overall, data from a total of 889 children were analyzed, with many studies involving a smaller number of subjects, and two including a larger study population [13,18]. The age of children exhibited a broad range of variation, from 1.2 years to 18 years. The total sample included 230 males (26%) and 629 females (70%), according to the female prevalence of Hashimoto’s thyroiditis, which is the main cause of acquired hypothyroidism [19]. One study did not report the sex distribution [22]. The sample population included children with autoimmune thyroiditis [12,13,16,17,18,19,21], not specified juvenile hypothyroidism [10,11,14,15,20], and subclinical hypothyroidism [14,22,23,25]. The follow-up in the studies was highly variable, spanning from a minimum of 6 months to a maximum of 11 years. Regarding the laboratory results, thyroid-stimulating hormone (TSH) and free thyroxine (fT4) values were reported in most studies, while free triiodothyronine (fT3) values were reported in fewer studies [10,15,17,20,25]. Anti-thyroglobulin (Tg-Ab) and anti-thyroperoxidase (TPO-Ab) antibodies were assessed in most studies. The levothyroxine (LT4) doses administered, when specified, varied across the different studies. However, in all instances, the dosages were within the ranges recommended by international guidelines. Regarding auxological parameters, the primary measure evaluated was the mean height standard deviation score (Ht-SDS), assessed both at the time of diagnosis and after a follow-up period. Additionally, two studies reported growth velocity in centimeters per year (cm/year) [15,22]. Parental height was analyzed in eight studies [10,12,14,16,18,19,21,24]. With respect to subclinical hypothyroidism [23,24,25], no significant effects on Ht-SDS were observed, except for the study by Cetinkaya et al. [22], in which all the children were below the first percentile for chronological age (Ht-SDS −2.79 ± 0.2 in the pre-pubertal group and −2.88 ± 0.2 in the pubertal group), with a bone age delay of two or more years compared to chronological age. In studies investigating acquired overt hypothyroidism, a lower mean Ht-SDS was reported at diagnosis in the majority of studies [10,14,15,16,17,18,19,20,21], and this deficit persisted after follow-up [10,11,13,14,16,19,21]. A summary of these results is reported in Table 3.

## 4. Discussion

Thyroid hormones are integral to numerous physiological processes, such as the regulation of energy expenditure, brain development, and skeletal growth [2,26]. In childhood and adolescence, these hormones are pivotal for physiological physical growth, particularly in maintaining a healthy height, as they are crucial for the proliferation and differentiation of chondrocytes: the cells responsible for bone growth in the growth plates (epiphyseal plates) of long bones [2,27,28]. In endochondral ossification, as seen in long bones, mesenchymal stem cells specialize into chondrocytes, which proliferate and create a cartilage matrix, forming a scaffold or anlage [29,30,31]. Chondrocyte hypertrophy, marked by an increase in cell volume within the epiphyseal ossification center and the organized, columnar alignment of these cells in the metaphyseal growth plate, has long been recognized as the fundamental structural mechanism driving longitudinal bone growth [32,33]. The ordered process of chondrocyte proliferation in the growth plate, hypertrophic differentiation, apoptosis, and subsequent new bone formation is essential for linear growth until a final height is reached [34]. This process is guided by different systemic hormones (including thyroid hormones), along with various cytokines and growth factors [34,35,36,37,38]. Therefore, hypothyroidism can have a profound impact on growth and development, particularly if it insidiously develops during early childhood, leading to delays in diagnosis and the initiation of replacement therapy [1].

Congenital hypothyroidism is currently diagnosed through newborn screening, which has significantly reduced the average age at diagnosis and improved both auxologic and neurological outcomes [39,40,41,42]. Children with congenital hypothyroidism identified via newborn screening and managed early with an adequately high daily dose of LT4 typically experience normal growth and attain a normal adult height [39,40,41,42,43,44,45,46,47]. The main determinants of final height in congenital hypothyroidism are the age at treatment initiation and treatment quality, maintaining TSH levels within the normal range [48,49,50]. Conversely, children with acquired hypothyroidism exhibit delayed somatic growth, resulting in a shorter stature compared to their peers, with a risk of a reduced final height. This is due to the fact that about 80% of affected children and adolescents are generally symptomless in the earlier stages, leading to delay in diagnosis [1,51,52]. The loss in height can be attributed to the considerable delay between the onset of growth slowdown and diagnosis of acquired hypothyroidism, with an average reported delay of 4.3 years [20]. This delay is largely due to insufficient regular growth monitoring and lack of awareness regarding the clinical and laboratory signs of hypothyroidism [53,54,55]. For instance, in the study by Rivkees et al., growth deceleration preceded the diagnosis of hypothyroidism by an average of 4.2 ± 1.3 years, with patients being under the third height percentile for their age at the time of diagnosis [10]. Similarly, in case series by Miyazaki et al. and Quintos et al., a short stature and history of chronic symptoms were observed, suggesting the presence of undiagnosed previous hypothyroidism [56,57].

Short stature, characterized as a child’s height falling below the third percentile or two standard deviations (SD) below the average height for age and gender, has been reported at the time of diagnosis in numerous studies [15,58]. This suggests a prior hypothyroid state, which leads to growth slowdown. The clinical and biochemical investigation by Kucharska et al. revealed that growth arrest was the most frequent sign (77%), with 38% of children showing a height below the third percentile [17]. Similarly, Becker et al. documented a growth delay in 75% of children, with 17% presenting a Ht-SDS below 2, and Vincent et al. reported a median Ht-SDS of −2.7, with a height loss of 2.5 standard deviation score (SDS) [19,21]. Compared to healthy controls, Saari et al. documented that children with hypothyroidism were significantly shorter, with a mean Ht-SDS deviation from target Ht-SDS (THDEVSDS) of −0.34 [18]. In the case-control study by Al-Omari and Omer, the hypothyroid group exhibited a lower mean height (128.3 ± 13.69 cm versus 143.3 ± 13.45 cm) [20]. Dujovne et al. discovered that children with goiters had better height at the time of diagnosis than those without goiters, with a mean Ht-SDS of 0.2 versus −2.42. This difference may be attributed to the presence of a clinical sign that suggested a thyroid disfunction, facilitating earlier identification and intervention [16,59]. In contrast, De Vries et al. found that children with Hashimoto’s thyroiditis exhibited mean Ht-SDS within the normal range: 0.67 for euthyroidism, −0.01 for compensated hypothyroidism, and −0.20 for non-compensated hypothyroidism. However, the Ht-SDS was significantly higher in the euthyroid patients compared to those with hypothyroidism, underscoring the negative impact of hypothyroidism on growth outcomes, irrespective of the presence of the same underlying condition [13]. Regarding the limited studies available on subclinical hypothyroidism, the mean Ht-SDS were found to be normal and within the target height range, indicating no significant effect on stature at the time of diagnosis [24,25]. These current data could be explained by the delay in the diagnosis of acquired hypothyroidism and the duration of thyroxine deficiency before treatment. Therefore, in the event of growth arrest or growth slowdown, it is essential to investigate the overall thyroid function in order to anticipate diagnosis and promptly start replacement treatment [60,61,62].

Thyroxine replacement induces rapid catch-up growth, marked by a height velocity exceeding the statistical norms for age and/or developmental stage over a specified period of time, following a temporary phase of growth suppression [10,63]. Its function is to bring the child closer to or under favorable conditions, fully aligned with their original pre-retardation growth trajectory [64,65,66]. Molecular investigations in animal models have demonstrated that catch-up growth occurs because growth-inhibiting conditions, such as hypothyroidism, maintain the restricted proliferative ability of growth plate chondrocytes by slowing down the typical process of growth plate aging. Once the growth-inhibiting condition disappears, the growth plates exhibit reduced senescence, allowing them to grow more rapidly than normal [67,68,69]. Catch-up growth may be partial, as bone age progresses more rapidly than height, particularly when hypothyroidism is diagnosed at or around the onset of puberty rather than during the prepubertal period [27]. This aligns with the results observed by Dujovne et al., who reported that pubertal children reached a significantly shorter final height compared to prepubertal children (mean Ht-SDS: −2.82 versus −1.52; *p* = 0.0311), and with those of Becker et al., in which 40% of children achieved complete catch-up growth under treatment, with significantly higher improvements in prepubertal children (median delta (Ht-SDS—Mid-parental Ht-SDS) + 0.5) compared to pubertal children (median delta (Ht-SDS—Mid-parental Ht-SDS) −0.2; *p* = 0.049) [16,19]. Therefore, in cases of lower catch-up growth, puberty plays a seminal role, as shown by the accelerated progression of skeletal maturation [70].

It is well-established that puberty and growth have a bidirectional relationship. Understanding a child’s growth patterns before puberty begins can help to estimate the timing of puberty. A child with delayed bone development will have more growth potential compared to a child whose skeletal age matches the chronological age (71–73). Therefore, shorter children with delayed bone age will typically experience a longer pre-pubertal growth phase than those without, which can help explain why they may eventually surpass their taller peers, even if the intensity of the pubertal growth spurt is similar. During puberty, the rate of growth in height rises significantly, reaching its peak during the adolescent growth spurt, and it ultimately contributes to approximately 20% of a person’s final adult height [71,72,73]. Future research is required to determine whether interventions aimed at delaying puberty to postpone epiphyseal closure could enable these patients to reach a normal adult stature [70]. Although some case reports have shown an improvement in height outcomes with the incorporation of gonadotropin-releasing hormone agonists to LT4 therapy, there is no evidence supporting the use of other treatments alongside LT4 [14,74,75,76]. Various hypotheses have been suggested to explain why pubertal children frequently do not achieve their genetic height potential, despite the notable catch-up growth seen with thyroid hormone replacement. It is suggested that the growth-inhibiting effects of sex steroids during puberty might be involved [14,77,78,79]. Furthermore, the initiation of therapy during puberty, later than the pre-pubertal period, may account for the failure to achieve a final height appropriate for age. Another factor that could affect growth is the severity of hypothyroidism at diagnosis, as demonstrated by Becker et al., who found that it was significantly correlated with fT4 and T4 levels [19]. Most studies have demonstrated a negative correlation between hypothyroidism and statural growth, with incomplete catch-up growth despite replacement therapy. Becker et al. reported that while most children exhibited some degree of catch-up growth, as revealed by a positive delta value in Ht-SDS, the majority still had a negative Ht-SDS value at the end of follow-up. This finding suggests that even adequate LT4 therapy cannot fully compensate for minor loss in height [19]. In the study by Vincent et al., the patients treated with LT4 showed an improvement of median Ht-SDS from −1.9 to −1.2 after one year, to −0.4 after two years, but growth decelerated during follow-up, with median heights of −0.5 at 3 years, −0.7 at 4 years, and −1.7 at 5 years. Nonetheless, the median final Ht-SDS was −1.4 [−2.7; 1.5], with a significant discrepancy between height loss at diagnosis and genetic target height, as well as overall catch-up growth [21]. This study highlighted inadequate catch-up growth, as the final height remained significantly lower than both the target Ht-SDS (1.5 [0.2; 2.7]) and the Ht-SDS before the onset of growth deflection (1.7 [0.2; 2.9]) [21]. In the study by Pantsiouou et al., all children failed to reach their genetic growth potential, with a final height below the mean for the normal population. This indicates that treatment with LT4 did not result in growth normalization or complete catch-up growth [11]. In contrast, de Vries et al. reported that 83% of children achieved their final height within both the normal and expected genetic ranges, possibly due to the shorter duration of hypothyroidism resulting from early detection and prompt treatment. On the other hand, Saari et al. observed full catch-up linear growth among children [13,18]. The efficacy of replacement therapy was further corroborated by Gutch et al., who observed an increase in Ht-SDS from −2.9 ± 0.9 at the initiation of LT4 therapy to −1.8 ± 0.8 after 12 months, with an increase in height velocity from 4.9 ± 0.8 cm/year in the year preceding treatment to 8.7 ± 1.3 cm/year during therapy [15].

Several hypotheses have been proposed to justify the incomplete catch-up growth. A prolonged deficiency in thyroid hormones may directly hinder the potential catch-up growth by causing molecular, functional, and structural damage to the epiphyseal growth plate. Additionally, inadequate treatment, either through underdosing or overdosing, may delay or accelerate bone maturation, respectively, potentially leading to premature epiphyseal fusion [21,80,81]. Furthermore, initiation of treatment during puberty could result in accelerated bone maturation before achieving an optimal prepubertal height, causing growth plate fusion prior to the completion of expected growth [21]. Another hypothesis involves concomitantly lower insulin-like growth factor 1 (IGF-1) levels, as reported by Vincent et al., suggesting a combined deficit of growth hormone (GH), IGF1, and peripheral hypothyroidism [21]. In fact, it is well-known that thyroid hormones not only cooperate but also drive GH secretion and IGF-1 production [82,83]. Previous studies by Chernausek et al. and Liu et al. demonstrated that GH secretion is significantly lower in children with hypothyroidism, and this reduction is reversible in many cases following treatment with thyroid hormones, although lower GH levels have been observed six months after starting of replacement treatment [84,85].

Regarding subclinical hypothyroidism, the limited available studies reported no significant alterations in growth, with no effects on Ht-SDS [22,23,24,25]. For instance, Cetinkaya et al. demonstrated that LT4 treatment in children with subclinical hypothyroidism could enhance growth velocity, with a significant improvement in height, although this should be compared to a control group that did not receive treatment [22].

The overall data from this review indicate that hypothyroidism significantly impacts height and growth outcomes, particularly when diagnosed later or during the pubertal phase. LT4 replacement therapy is essential to reduce the risk of a final short stature, although this may not lead to complete catch-up growth.

## 5. Limitations

Although our review summarizes the most recent available findings on the effects of hypothyroidism on children’s height, it has several limitations. Firstly, the screening process may not be flawless, and pediatric studies not indexed in PubMed or those not identified through our keyword searches could have been missed. Therefore, larger-scale studies are necessary in the future to better explore this relationship. Lastly, we did not conduct a meta-analysis due to the variability among the included studies, especially regarding sample size, children’s age, auxological parameters, and follow-up duration.

## 6. Conclusions

In conclusion, thyroid hormones have a crucial role in growth and development, particularly during childhood, when they are essential for normal skeletal growth. Hypothyroidism, especially if diagnosed late or during puberty, can significantly impair growth, leading to shorter stature and increased risk of reduced final height. Our review highlights the need for early diagnosis and timely initiation of LT4 replacement therapy to mitigate these effects. While LT4 treatment usually determines catch-up growth, particularly in younger children, it may not fully normalize growth in all cases, mostly when hypothyroidism is diagnosed during puberty. Ultimately, careful growth monitoring and increased awareness of hypothyroidism symptoms are essential for minimizing growth deficits and optimizing long-term outcomes for children with hypothyroidism.

## Figures and Tables

**Table 1:** List of studies related to overt acquired hypothyroidism.

Study	Design	Sample Size (N)	Mean Age at Diagnosis (Years)	M:F	Patient Population	L-Thyroxine Median Dosage	Growth Measures
Rivkees et al., 1988 [10]	Clinical study	24	11.4 ± 2.7 years	6:18	Juvenile hypothyroidism	3.4 ± 0.3 μg/kg per day	At diagnosis: females 4.04 ± 0.5 SD and males 3.15 ± 0.4 SD under the mean heights for age. Adult heights were 2 SD lower than normal: 149 ± 5.0 cm for females and 168 ± 5.1 cm for males (lower than mid-parental heights and pre-illness HtSDS).
Pantsiouou et al., 1991 [11]	Clinical study	29	8.8 (F) 9.5 (M)	9:20	Juvenile hypothyroidism	100 μg/m^2^/day	In females, the mean HtSDS for bone age was +0.59 before treatment and −0.55 after eleven years of treatment. In males, the mean HtSDS for bone age was + 1.6 before treatment and −0.87 after nine years of treatment.
Jaruratanasirikul et al., 2001 [12]	Clinical study	46	12.4 ± 1.7	3:43	Hashimoto’s thyroiditis	2 μg/kg/day	The final adult stature was 0.43 ± 0.80 SDS, which was 1.58 ± 3.03 cm above MPH.
De Vries et al., 2009 [13]	Retrospective study	114	11.8	22:92	Hashimoto’s thyroiditis	1.5 μg/kg/day	At diagnosis: mean HtSDS of 0.67 for euthyroidism, −0.01 for compensated hypothyroidism, −0.20 for non-compensated hypothyroidism. At the last visit (6 years), HtSDS was significantly smaller than at diagnosis for the whole study group (−0.45 vs. −0.05).
Nebesio et al., 2011 [14]	Comparative study	21	10.1 ± 3.0	6:15	Juvenile hypothyroidism	22.6 ± 6.4 μg/day	At diagnosis: HtSDS—3.0 ± 1.1 (range of −0.9 to −5.0). Thirteen patients reached FAH, of which six received adjunctive GPTs. No differences in height changes from diagnosis to FAH. between those who received GPTs and those who did not (mean change in height SDS was −1.1 vs. −1.6). In both groups, the patients were significantly shorter than mid-parental height, at baseline and at FAH.
Gutch et al., 2014 [15]	Clinical study	87	11.2 ± 2.3	27:60	Juvenile hypothyroidism	NA	HtSDS −2.9 ± 0.9 before the replacement therapy. HtSDS from −2.9 ± 0.9 at the start of thyroxine treatment to −1.8 ± 0.8 after 1 year of treatment (*p* < 0.001). Height velocity rose from 4.9 ± 0.8 cm/year in the year before treatment to 8.7 ± 1.3 during treatment (*p* < 0.001).
Dujovne et al., 2019 [16]	Retrospective study	79	10.9	17:62	Hashimoto’s thyroiditis	NA	Mean HtSDS 0.2 in children with goiters vs. −2.42 in children without. Children with short stature before puberty: height at the time of diagnosis −3 ± 0.28 vs. final stature −1.52 ± 0.21 Children with normal stature before puberty: height at the time of diagnosis −1.07 ± 1.27 vs. final stature −1.71 ± 1.37 Children with short stature during puberty: height at the time of diagnosis −3.72 ± 0.77 vs. final stature −2.82 ± 1.31 Children with normal stature during puberty: height at the time of diagnosis 0.31 ± 1.09 vs. final stature −0.6 ± 1.09. Mean final HtSDS −2.82 in pubertal children vs. −1.52 in prepubertal ones.
Kucharska et al., 2020 [17]	Retrospective study	26	10.26 ± 3.3	9:17	Hashimoto’s thyroiditis	NA	Growth arrest was the most common sign (77%), while absolute short height (<3rd percentile for Polish population) was only recorded in 38%.
Saari et al. 2021 [18]	Retrospective study	109	10.6	27:72	Hashimoto’s thyroiditis	NA	At diagnosis: children were considerably shorter than in the healthy control group (mean adjusted THDEVSDS difference, −0.34 [95% CI: −0.57 to −0.10). After 1 year of levothyroxine treatment: complete catch-up in linear growth and achievement of the target stature.
Becker et al., 2022 [19]	Retrospective study	43	10.6	5:38	Hashimoto’s thyroiditis	NA	At diagnosis: 75% growth delay (median HtSDS −0.55); 17% HtSDS < −2; 47% growth retardation based on growth charts. Following a median observation period of 3.4 years (0.6–10.3 years) of 26 children undergoing LT4 therapy: 40% achieved complete catch-up growth, which was significantly higher in prepubertal [median delta (height SDS-MPH SDS) + 0.5] than in pubertal children (median delta (height SDSMPH SDS) −0.2). Eighteen children had a negative height SDS-MPH SDS value.
Al-Omari et al., 2023 [20]	Case-control study	90	10.6 ± 3.01	38:52	Juvenile hypothyroidism	NA	The mean ±SD was 143.3 ± 13.45 cm in the control group compared to the group with short height (128.3 ± 13.69 cm).
Vincent et al., 2023 [21]	Multicenter study	29	9.7	14:15	Hashimoto’s thyroiditis	2.2 μg/kg/day	At diagnosis: median HtSDS −2.7 [−4.6; −0.1], with height loss of 2.5 [0.7; 5.4] SDS. After a median observational period of 66 months (5 years and 6 months) ± 43 months (3 years and 7 months) under LT4 therapy: median HtSDS increased from −1.9 SDS to −1.2 SDS at 1 year; to −0.4 SDS at 2 years, and then growth diminished during follow-up, with heights of −0.5 SDS at 3 years, −0.7 SDS at 4 years, and −1.7 SDS at 5 years. Median final height was −1.4 [−2.7; 1,5] SDS, with a significant discrepancy between height loss at diagnosis and genetic target height and total catch-up growth (*p* = 0.003).

Standard deviation (SD), height standard deviation score (HtSDS), mid-parental height (MPH), final adult height (FAH), growth-promoting therapies (GPTs), height-for-age deviation from the target height (THDEVSDS), mid-parental height standard deviation score (MPH-SDS), levothyroxine (LT4).

**Table 2:** List of studies related to subclinical acquired hypothyroidism.

Study	Design	Sample Size (N)	Mean Age at Diagnosis (Years)	M:F	Growth Measures
Cetinkaya et al., 2003 [22]	Clinical study	39	9.05 ± 2.0 (prepubertal) 13.15 ± 1.5 (pubertal)	NA	At diagnosis in the prepubertal group: height 116.94 ± 10.01 cm, HtSDS = −2.79 ± 0.2 At diagnosis in the pubertal group: height 136.97 ± 8.2 cm, HtSDS = −2.88 ± 0.2 After starting replacement treatment: (1) In children before puberty: GV 4.51 ± 1.8 cm at the beginning, 5.65 ± 2.7 cm after 6 months, and 5.53 ± 1.5 cm at one year. GVSDS increased from −1.29 ± 2.1 to −0.25 ± 2.8 and to 0.19 ± 1.52 after 6 months and 1 year. (2) In children during puberty: GV 5.54 ± 1.5 cm at the beginning, 8.26 ± 4.5 cm after 6 months, and 7.95 ± 2.81 cm at one year. GVSDS increased from −0.16 ± 2.4 to 4.02 ± 5.6 and 3.83 ± 3.7 after 6 months and 1 year.
Wasniewska et al., 2009 [23]	Prospective study	92	8.1 ± 3.0	20:42	No significant changes in the average HtSDS (−0.1 ± 1.3) at the end of follow-up with respect to study entry.
Cerbone et al., 2011 [24]	Clinical study	36	9.7 ± 0.6	19:17	At diagnosis: mean HtSDS normal (−0.8 ± 0.2 SDS) and within the target height (−1.2 ± 0.2 SDS). After an observational period of 3.3 ± 0.3 years: no decline with respect to height (−0.7 ± 0.2 SDS).
Ergin et al. 2018 [25]	Clinical study	25	9.65 ± 2.89	8:17	HtSDS −0.71 ± 0.87 at diagnosis, −0.69 ± 0.74 at the third month, −0.68 ± 0.79 at 1 year of follow-up. No effects on HtSDS.

Height standard deviation score (HtSDS), growth velocity (GV), growth velocity standard deviation score (GVSDS).

**Table 3:** Summary of the literature research performed in this study.

Year of publication	1988–2023
Mean age of children	1.2 years–18 years
Sex distribution	230 males (26%), 629 females (70%)
Follow-up duration	6 months–11 years

## Data Availability

Not applicable.
